# Molecular Network Associated with MITF in Skin Melanoma Development and Progression

**DOI:** 10.1155/2011/730170

**Published:** 2011-10-20

**Authors:** Ichiro Yajima, Mayuko Y. Kumasaka, Nguyen Dinh Thang, Yuji Goto, Kozue Takeda, Machiko Iida, Nobutaka Ohgami, Haruka Tamura, Osamu Yamanoshita, Yoshiyuki Kawamoto, Keiko Furukawa, Masashi Kato

**Affiliations:** ^1^Unit of Environmental Health Sciences, Department of Biomedical Sciences, College of Life and Health Sciences, Chubu University, Kasugai-shi, Aichi 487-8501, Japan; ^2^Unit of Immunology, Department of Biomedical Sciences, College of Life and Health Sciences, Chubu University, Kasugai-shi, Aichi 487-8501, Japan; ^3^Health Science Hills, College of Life and Health Sciences, Chubu University, Kasugai-shi, Aichi 487-8501, Japan; ^4^Unit of Biochemistry, Department of Biomedical Sciences, College of Life and Health Sciences, Chubu University, Kasugai-shi, Aichi 487-8501, Japan

## Abstract

Various environmental and genetic factors affect the development and progression of skin cancers including melanoma. Melanoma development is initially triggered by environmental factors including ultraviolet (UV) light, and then genetic/epigenetic alterations occur in skin melanocytes. These first triggers alter the conditions of numerous genes and proteins, and they induce and/or reduce gene expression and activate and/or repress protein stability and activity, resulting in melanoma progression. *Microphthalmia-associated transcription factor* (*MITF*) is a master regulator gene of melanocyte development and differentiation and is also associated with melanoma development and progression. To find better approaches to molecular-based therapies for patients, understanding MITF function in skin melanoma development and progression is important. Here, we review the molecular networks associated with MITF in skin melanoma development and progression.

## 1. Introduction

Much evidence that environmental factors are correlated with various diseases has been accumulating. The environmental factors can be classified into physical [1–4], chemical [5–8], and biological [9, 10] factors. In addition to the environmental factors, genetic factors also have a great influence on the development and pathogenesis of various diseases [2, 11, 12]. Skin is a representative organ that directly suffers from environmental factors. There is much evidence showing that sunlight and ultraviolet light induce various skin cancers with modulation of the signaling of cell proliferation and DNA damage [13–16]. Therefore, roles of skin cancer-related molecules should be discussed with consideration of the effects of environmental factors. Moreover, the incidence of skin melanoma has recently been increasing at a greater rate than that of any other cancer [17]. In the USA, 68,130 cases of invasive melanoma and at least 48,000 cases of melanoma *in-situ* were diagnosed in a year [18]. Since melanoma is the most aggressive skin cancers [17, 18], we focus on skin melanoma in this paper.

 Not only studies on humans including epidemiological research but also animal models can be useful for analyzing melanomagenesis [19–23]. For example, exposure of skin to oxygen might regulate development of benign melanocytic tumors with modulation of tumor immunity in animal models [19]. Ultraviolet (UV) light is correlated with malignant transformation from benign melanocytic tumors and melanoma [15, 24]. In addition to these environmental factors, various kinds of genetic factors have been reported as crucial factors of melanoma. For example, tyrosine kinases are important for the development and pathogenesis of melanoma in mice and humans [19, 21–23, 25–27]. Various membrane trafficking-associated molecules have also been reported to be involved in melanoma pathogenesis [28–30]. Moreover, some acidic glycosphingolipids have been reported to be expressed at high levels in melanomas and promote their malignant properties by activating cell growth and adhesion signals in melanoma cells [31–33]. 

 Microphthalmia-associated transcription factor (MITF) is believed to be one of the master molecules to regulate melanomagenesis among the many previously reported melanoma-associated molecules. Therefore, we selected MITF as a cancer-associated molecule in melanoma and introduce recent findings regarding MITF in this paper.

## 2. Results

Melanocytes, melanin-producing cells that are widely distributed in several tissues from fungi to primates on the long evolutionary process, have multifunctionality for survival strategy [34–41]. Melanocytes are also present in skin surfaces and protect them from UV that damages DNA, thereby causing genotoxic mutations or skin cancers [42, 43], but once they transform, they can result in the development of one of the most serious cancers, melanoma.

### 2.1. Microphthalmia-Associated Transcription Factor

**Figure 1:**
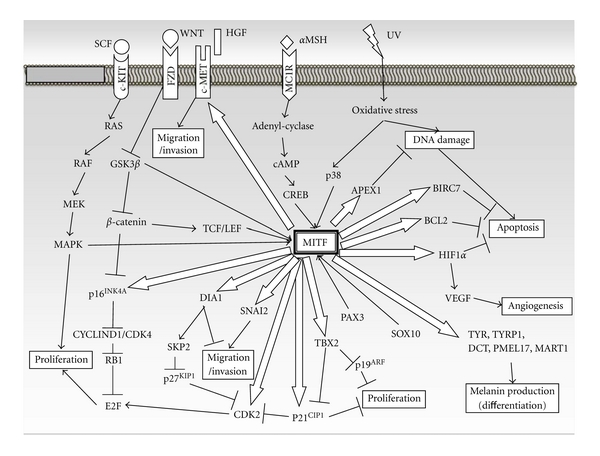
MITF-centered schematic scheme of cellular signaling on melanoma development and progression. Open arrows indicate direct transcriptional targets of MITF. The amount of transcripts of these targets is regulated by direct MITF binding to *cis*-elements in their promoter sequence. Black arrows or lines indicate signal cascades associated with melanoma development and progression. Arrows or lines toward MITF mean direct association by binding to the *MITF* promoter region or MITF protein. *α*MSH: melanocyte-stimulating hormone; BCL2: B-cell leukemia/lymphoma 2; BRN2: brain-2; CDK2/CDK4: cyclin-dependent kinase 2/4; CREB: cAMP-responsive element-binding protein; DCT: dopachrome tautomerase; DIA1: diaphanous homolog 1; FZD: frizzled; GSK3*β*: glycogen synthase kinase 3 beta; HGF: hepatocyte growth factor; HIF1*α*: hypoxia inducible factor 1, alpha subunit; MC1R: melanocortin 1 receptor; MDM2: transformed mouse 3T3 cell double minute 2; PMEL17: premelanosome protein; RB1: retinoblastoma 1; SCF: stem cell factor; SNAI2: snail homolog 2; SOX10: Sry-related HMG box 10; TYR: tyrosinase, WNT: wingless-related MMTV integration site.

The *MITF *gene, encoding a basic-helix-loop-helix-leucine zipper transcription factor, is expressed in melanocytes, retinal pigmented epithelium, mast cells, osteoclasts, and melanoma [36, 37, 40–42, 44–49]. MITF protein forms dimers and binds to specific consensus DNA sequences in the promoter regions of various target genes to regulate several events including differentiation, proliferation, migration, invasion, and tumorigenesis (Figure 1) [50]. 

### 2.2. Regulation of MITF Expression and Activity

Several transcription factors directly control *MITF* gene transcription to regulate melanocyte and melanoma development. Paired box 3 (PAX3) and Sry-related HMG box 10 (SOX10), highly correlated with melanocyte development and melanomagenesis [51–53], positively regulate *MITF* expression by directly binding to* MITF* promoter regions [54–57]. Activation of melanocortin 1 receptor (MC1R) by binding of alpha-melanocyte stimulating hormone (*α*-MSH) induces cAMP production via activation of adenylyl cyclase and phosphorylates cAMP response element-binding protein (CREB). Phosphorylated CREB directly binds to the *MITF* promoter region and stimulates *MITF* transcription [58, 59]. Wingless-type (WNT) signaling is often activated in human melanoma [60–63]. Activation of Frizzled receptors by binding of WNT molecules enhances interaction of *β*-catenin with TCF/LEF transcription factors, resulting in stimulation of *MITF* promoter activity [64–66].

Furthermore, MITF protein is modified by several factors after translation. Phosphorylation at Ser 301 of MITF is induced by UV through p38 stress-activated kinase [67], and Ser 298 of the protein is phosphorylated by GSK3*β* [65], resulting in stimulation of MITF transcriptional activity. The c-KIT receptor activated by stem cell factor (SCF, c-KIT ligand) phosphorylates Ser 73 and thereby increases MITF transcriptional activity followed by immediate degradation of MITF [59, 68], whereas sumoylation at Lys 182 and Lys 316 increases MITF transcriptional activity [69, 70].

### 2.3. Transcriptional Targets of MITF

MITF is associated with cellular senescence, apoptosis, proliferation, migration/invasion, and differentiation through regulating transcription of target genes.

Overcoming cellular senescence, acquisition of anti-apoptotic activity, and promotion of proliferation are critical cellular events for the initiation of tumorigenesis [50, 71–75]. *CDKN2A* and *WAF1* genes encode senescence mediator proteins, *p16* ^*INK4A *^ and *p14* ^*ARF *^, and *p21* ^*Cip1 *^, respectively, and are the well-known familial melanoma locus [71, 73, 76]. Copy number of* CyclinD1* (*CCND1*) gene, a cell cycle mediator, is amplified in 25% of human melanomas [77]. MITF directly binds to the promoter regions of *p16* ^*Ink4a *^, *p21* ^*Cip1 *^, and *CyclinD1* and positively regulates their transcription [78–80]. T-Box transcription factor 2 (TBX2) is highly expressed in melanoma cell lines and represses *p19* ^*ARF *^ and *p21* ^*Cip1 *^, both of which are implicated as effectors of senescence, promotes proliferation, and suppresses senescence in melanoma [81, 82]. *TBX2* has also been described as one of the MITF target genes [81]. These reports indicate that MITF is linked to melanoma development as a transcriptional activator of senescence-/proliferation-associated genes.

Antiapoptotic effect is a key process for melanoma development. *B-cell leukemia/lymphoma 2 (BCL2)* is an antiapoptotic gene and is widely expressed in human melanomas [83–85]. *BCL2* is an MITF target gene and is activated at the transcription level [86]. Baculoviral IAP repeat containing 7/melanoma inhibitor of apoptosis (BIRC7/ML-IAP), which is an antiapoptotic regulator, is highly expressed in human melanomas [87] and provides resistance to apoptosis-based chemotherapeutic treatments [88]. *BIRC7* transcription is also directly activated by MITF, and overexpression of BIRC7 rescued melanoma from apoptosis in MITF-depleted melanoma cells [87]. Antioxidative stress activity is important for melanocyte survival and melanoma development. Oxidative stress from environmental factors such as solar UV causes DNA damage and apoptosis. Recently, *apurinic/apyrimidinic endonuclease1/redox factor-1 *(APEX1/Ref1) has been identified as a MITF target gene and has been shown to be partially rescued from oxidative stress-induced apoptosis in MITF-depleted cells [89]. *Hypoxia-inducible factor 1 *α* (HIF1*α*)* has also been demonstrated to be activated at the transcription level by direct MITF binding to the *HIF1*α** promoter region and acts as an antiapoptotic factor in melanoma cells [90]. Antiapoptotic activity and resistance to chemotherapy of melanoma are under the control of MITF activity.

Angiogenesis and invasion are critical steps for tumor progression, and these activities are enhanced in melanoma. MITF depletion in melanoma cells represses not only transcription of *HIF1*α** but also that of vascular endothelial growth factor (*VEGF*), which is a target of HIF1*α* and has been demonstrated to be a major contributor to angiogenesis [90]. The *c-MET* protooncogene, which encodes hepatocyte growth factor receptor (HGFR), is highly expressed in human melanomas and linked to metastatic potential in melanomas. *c-MET* transcription is directly regulated by MITF [91]. SNAI2 has been reported to be a key player for epithelial-mesenchymal transition (EMT), which is a crucial phenomenon during invasion, metastasis of melanoma, by repressing *E-cadherin* transcription and stimulating fibronectin expression, and MITF directly activates the transcription level of *SNAI2* [92–94]. On the other hand, MITF directly binds to the promoter region of *diaphanous homolog 1 (DIAPH1, DIA1)* gene and activates its transcription, resulting in inhibiting the invasiveness of melanoma by activation of actin polymerization [95].

MITF is also well known as a master regulator of melanin production. Melanin pigment is synthesized from tyrosine via an enzymatic process. This process is catalyzed by tyrosinase family proteins, tyrosinase (TYR), tyrosinase-related protein 1 (TYRP1), and DCT (dopachrome tautomerase). After melanin production, melanin pigment is stored in melanosomes, which are organelles containing melanin, and is transported to the skin for UV protection. MART1 and PMEL17 are localized in melanosomes and contribute to melanosome maturation [96–99]. Direct regulation of melanin synthesis-associated genes at transcription levels by MITF stimulates melanin production. [100–103].

### 2.4. “Two-Faced” Function of Mitf in Melanoma Development and Progression

MITF is expressed in most human melanomas, and stability of its expression is essential for melanoma cell proliferation and survival [104]. In addition, amplification of the *MITF* locus was observed in human metastatic melanomas [105]. However, the expression level of MITF in melanomas is significantly lower than that in normal melanocytes, and higher expression level of MITF in melanoma represses cell proliferation even in the presence of oncogenic BRAF [106]. Most likely, MITF plays both cancer-promoting and cancer-inhibiting roles alternated by the expression level and/or activity. A low level of MITF expression promotes proliferation in melanoma, whereas a high level of MITF expression promotes differentiation through induction of cellular senescence and melanin production [107–109]. MITF has the ability to upregulate transcription of melanoma-promoting genes (e.g., *CyclinD1*, *BCL2*, *c-MET*) and also that of melanoma-repressing genes (e.g., *p16* ^*Ink4a *^, *p21* ^*Cip1 *^, *DIAHP1*). Taken together, MITF has two-faced functions in melanoma development and progression, and strict regulation of MITF in its expression and/or activity is likely to switch melanocytes to melanoma cells.

## 3. Concluding Remarks

Skin melanoma is an aggressive tumor of the skin, and patients have a poor prognosis. Analysis of the expression profile and function of MITF and identification of its target genes are important to better understand the complex system of melanoma development and progression. Expression patterns, functions, and many target genes of MITF have been reported by a number of groups, though the complicated functions of MITF in skin melanoma development and progression are still not well understood. Extensive analyses of MITF will lead to a better understanding of melanoma development and progression and to the establishment of more effective therapeutics.
